# Commentary: First application of whole blood exchange-lymphoplasmapheresis combined transfusion for restoring immune homeostasis of ceftriaxone-induced hemolytic crisis: a case report

**DOI:** 10.3389/fimmu.2026.1953665

**Published:** 2026-09-15

**Authors:** Jinyang Bu, Xinfu Zou, Aojiao Chu, Ling Shi, Meihong Luo

**Affiliations:** Department of Hematology, Baoshan Hospital, Shanghai University of Traditional Chinese Medicine/Shanghai Baoshan Hospital of Integrated Traditional Chinese and Western Medicine, Shanghai, China

**Keywords:** ceftriaxone, drug-induced immune hemolytic anemia, hemolytic crisis, therapeutic apheresis, whole blood exchange

## Introduction

We read with great interest the case reported by Shen et al. (1), in which a 57-year-old woman developed shock, dark-brown urine, and a precipitous fall in hemoglobin shortly after ceftriaxone administration. A strongly positive direct antiglobulin test and ceftriaxone-dependent IgG and IgM antibodies supported the diagnosis of ceftriaxone-induced drug-induced immune hemolytic anemia (DIIHA). Following ceftriaxone withdrawal, intensive supportive care, transfusion, glucocorticoids, two sessions of whole blood exchange-lymphoplasmapheresis combined transfusion (WLCT), and subsequent dual plasma molecular adsorption, the patient recovered. Because DIIHA is rare but may cause fulminant intravascular hemolysis and organ injury within hours, the report addresses an important rescue-treatment gap (2–4, 6).

## Mechanistic rationale and evidentiary boundaries

The principal value of this report is its mechanism-oriented treatment concept. Ceftriaxone withdrawal prevents renewed drug exposure, whereas WLCT is intended to reduce circulating drug-dependent antibodies, sensitized or damaged red blood cells (RBCs), and a cellular fraction that may contain antibody-producing cells. This integrated target is biologically attractive because therapeutic plasma exchange mainly removes soluble plasma constituents, while RBC exchange mainly replaces circulating RBCs. Drug-dependent antibody testing also provides stronger causal support than temporal association alone.

However, the phrase “restoring immune homeostasis” should be interpreted cautiously. Falling antibody titers, resolution of hemolysis, and conversion of the direct antiglobulin test to negative demonstrate improvement in the intravascular immune-hematologic state, but do not establish systemic immune reprogramming. Removal of antibody-producing cells was not directly quantified, and changes after WLCT may reflect a combination of cell removal, plasma dilution, transfusion, natural antibody clearance, and cessation of ceftriaxone exposure. Pre- and post-procedure measurements of recovered cell subsets, circulating plasmablasts, complement activation, and standardized drug-dependent antibody titers would clarify which components WLCT actually modifies.

## Clinical positioning, safety, and reproducibility

The therapeutic contribution of WLCT cannot be isolated from this single case. Drug withdrawal, anti-shock care, glucocorticoids, RBC and plasma transfusion, two WLCT procedures, and dual plasma molecular adsorption were delivered within a short interval. The favorable outcome therefore supports feasibility and a temporal association, not comparative efficacy. Immediate and permanent ceftriaxone withdrawal, transfusion support, and organ-directed critical care remain the foundation of management; WLCT should be presented as an investigational rescue option for selected patients with ongoing life-threatening hemolysis, shock, or evolving organ injury despite initial measures.

Reproducibility and safety also require attention. The reported procedure depended on manual separator adjustment and visual assessment of collection-line color. A transferable protocol should specify patient selection, exchange volume, replacement fluid and blood-component ratios, anticoagulation and calcium management, device settings, stopping rules, and surveillance for hemodynamic instability, citrate toxicity, bleeding, vascular-access complications, and transfusion reactions (5). A multicenter registry could use harmonized diagnostic criteria and fixed-time-point measurements of hemoglobin, bilirubin, lactate dehydrogenase, haptoglobin, plasma-free hemoglobin, renal function, complement, antibody titers, vasopressor exposure, transfusion requirements, and adverse events.

## Discussion

Shen et al. should be commended for describing a creative, pathophysiology-oriented intervention for an immediately life-threatening immune adverse drug reaction. The report does not establish WLCT as standard therapy, but it generates a clinically relevant hypothesis: combined removal of soluble antibodies and sensitized cellular components may offer rapid control when conventional supportive measures are insufficient. Standardized procedures, direct mechanistic measurements, and multicenter comparative data are now needed to determine whether this promising rescue concept is safe, reproducible, and associated with better patient-centered outcomes.
